# Automated Delineation of Vessel Wall and Thrombus Boundaries of Abdominal Aortic Aneurysms Using Multispectral MR Images

**DOI:** 10.1155/2015/202539

**Published:** 2015-07-05

**Authors:** B. Rodriguez-Vila, J. Tarjuelo-Gutierrez, P. Sánchez-González, P. Verbrugghe, I. Fourneau, G. Maleux, P. Herijgers, E. J. Gomez

**Affiliations:** ^1^Biomedical Engineering and Telemedicine Centre, ETSI de Telecomunicación, Universidad Politécnica de Madrid, 28040 Madrid, Spain; ^2^Networking Research Centre on Bioengineering, Biomaterials and Nanomedicine (CIBER-BBN), 28040 Madrid, Spain; ^3^Laboratory of Experimental Cardiac Surgery, Department of Cardiovascular Diseases, Gasthuisberg University Hospital, University of Leuven, 3000 Leuven, Belgium

## Abstract

A correct patient-specific identification of the abdominal aortic aneurysm is useful for both diagnosis and treatment stages, as it locates the disease and represents its geometry. The actual thickness and shape of the arterial wall and the intraluminal thrombus are of great importance when predicting the rupture of the abdominal aortic aneurysms. The authors describe a novel method for delineating both the internal and external contours of the aortic wall, which allows distinguishing between vessel wall and intraluminal thrombus. The method is based on active shape model and texture statistical information. The method was validated with eight MR patient studies. There was high correspondence between automatic and manual measurements for the vessel wall area. Resulting segmented images presented a mean Dice coefficient with respect to manual segmentations of 0.88 and a mean modified Hausdorff distance of 1.14 mm for the internal face and 0.86 and 1.33 mm for the external face of the arterial wall. Preliminary results of the segmentation show high correspondence between automatic and manual measurements for the vessel wall and thrombus areas. However, since the dataset is small the conclusions cannot be generalized.

## 1. Introduction

An abdominal aortic aneurysm (AAA) is a pathological dilation in a segment of the abdominal aorta, where the aortic diameter is greater than 3 cm or 50% greater than the uninvolved proximal vessel [1]. One of the biggest risks associated with this disease is the weakening of the aortic wall, which can lead to dissection or rupture of the artery. Blood stagnation is also possible in the dilation, inducing formation of an intraluminal thrombus (ILT) [2].

Recent progress in medical imaging supports clinicians in diagnosis and subsequent treatment of AAA in different stages. New technologies and methods in magnetic resonance imaging (MRI) as well as computed tomography angiography (CTA) have enhanced soft tissue contrast and enabled clinicians to distinguish between the soft tissue structures of interest [1]. Different acquisition protocols, especially in MRI, open the possibility of differentiating between the AAA wall and the intraluminal thrombus within the context of AAA imaging.

Amongst other important physical and physiological variables, outer wall boundary delineation eases vascular treatment planning. Moreover, to measure actual vessel wall thickness may result in more accurate mathematical models for rupture risk prediction [3].

There are a significant number of studies in the state of the art regarding the segmentation of vascular structures. Specifically, for segmentation of AAA from medical images, commonly CTA and magnetic resonance angiography (MRA) are employed. Many different techniques may be applied in order to perform the segmentation of AAAs that can be classified according to the kind of information that guides the segmentation process. Thus, there are methods based on the raw intensity information of the image, such as clustering [4], multiscale [5], or histogram information [3, 6]. There are methods based on the information provided by the intensity gradient that is used to control a deformable model. Such well-known techniques are active contours [7–9], level sets [10–13], and graph search [14]. Finally, there are techniques based on statistical models guided by a priori information. This information is extracted from a controlled and manually processed set of images known as training set. The most common statistical models for medical imaging processing are the active shape models (ASM) [15] and the active appearance models (AAM) [16], which may be used to segment AAAs [17–19].

Most of the studies in the literature regarding AAA segmentation only consider the outer face of the aortic wall. Being so, it is not possible to distinguish between the wall and the ILT, which are differentiated conforming structures. To the authors' knowledge, only Zohios et al. [13] addressed the segmentation of both the inner and the outer face of the aortic wall. However, this work is limited to patient studies that present calcifications in 3D CTA scans and their evaluation stage was very limited in the case of the thrombus segmentation.

We present a modular method, based on a statistical shape model and texture information, for segmenting human AAA geometries in MR multispectral studies. The proposed method allows quantitative measurements of morphological aspects useful for treatment planning and may lead to more accurate methods for the evaluation of their biomechanical environment.

Although each patient's aneurysm is unique, characterized by its location and shape, and must be accurately represented for subsequent analyses to be meaningful, about 90% of AAAs are located below the renal arteries [1]. Thus, our investigation focuses on modelling the lower part of the aorta between the renal arteries and the aortic bifurcation into the iliac arteries.

## 2. Materials and Methods

### 2.1. Patient Images

Different MRI acquisition protocols open up the possibility of discriminating between different soft tissue structures. We developed an MRI protocol focused on providing high contrast for the AAA wall and the ILT against the surrounding soft tissue. If no contraindications existed, we performed MRI studies using 1.5 T Aera scanner (Siemens, Erlangen, Germany) on patients with AAAs of diameter larger than 5 cm. We registered the MRI study at the clinical trial centre of the University Hospital of Leuven (study number S52774) and obtained ethical approval from the ethical committee at UZ Leuven.

Regarding the MRI data, we used a sagittal and transversal balanced steady-state free precession (bSSFP) sequence as a localizer, with 20 sagittal slices of 5 mm slice thickness and 30 transversal slices of 6 mm slice thickness, both with no intersection gap, a field of view (FOV) of 380 mm, a matrix size of 320 × 260, a time to repetition/time to echo (TR/TE) of 4.41 ms/2.21 ms, a flip angle of 62°, and a one signal average. Thereafter, we completed a pulse triggered, three-slice T1 Turbo Spin Echo (TSE) sequence with 6 mm slice thickness, TR/TE of 800 ms/62 ms, FOV of 160 mm, and a matrix of 256 × 256 and a flip angle of 180°. Next, we performed a coronal breath-hold fast low-angle shot (FLASH) 3D sequence after intravenous administration of a standardized dose of 0.1 mmol/kg Gd-DOTA (Dotarem, Guerbet, France). We executed this in the arterial phase with a slab thickness of 96 slices of 1.25 mm, 384 × 336 matrix size, TR/TE of 3.04 ms/1.09 ms, and a FOV of 400 mm and a flip angle of 25°.

**Figure 1:**
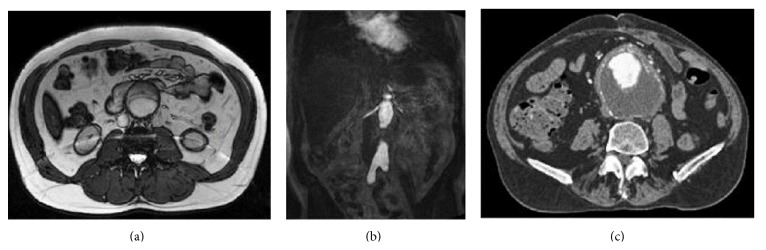
(a) MR transversal bSSFP for thrombus and outer wall segmentation; (b) MR coronal FLASH for lumen segmentation; (c) CTA image for shape modelling.

After acquisition, we evaluated image quality and tissue contrast for the AAA wall and the ILT in order to select images which best met our objectives. After analysis, we selected the transversal bSSFP sequence for segmentation of ILT and aortic wall (Figure 1(a)), and coronal FLASH contrasted sequence for lumen segmentation (Figure 1(b)).

The main drawback of this kind of images is that they are not currently in clinical use. This makes it difficult to obtain an ample image dataset, and thus only 8 patients were included in the experiment dataset. Due to this limitation, and since a higher number of cases are needed to characterize the shape variations, 75 CTA images of AAA patients were used to characterize the possible shape configurations of the infrarenal abdominal aorta in a more robust way (Figure 1(c)). The studied section of the aorta is the lower 12 cm, just above the bifurcation of the aorta into the iliac arteries, and always below the renal arteries. The CTA images were cropped and resampled to images of 512 × 512 × 19 voxels, with dimensions of 1 × 1 × 0.66 mm.

### 2.2. Proposed Segmentation Algorithm

**Figure 2:**
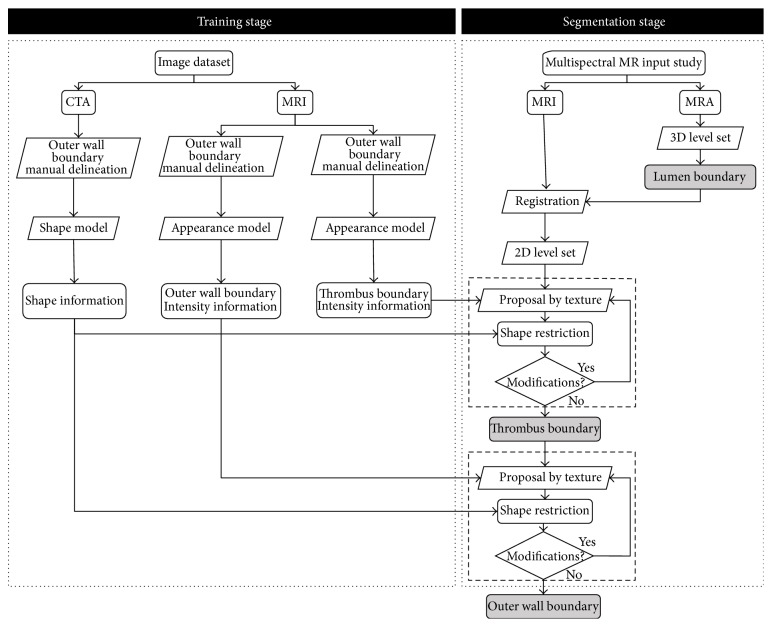
Diagram of the proposed segmentation algorithm. The initial training stage guides the segmentation process from the inner structures (lumen), through the thrombus, to the outer wall boundary.

The basic steps of the segmentation algorithm proposed in this work are shown in Figure 2. In a first training stage, the system extracts texture and shape information to guide the segmentation process. Outer wall boundary is manually delineated on the CTA images and an active shape model (ASM) is constructed using the segmented images. The texture information characterizes the intensity properties of the target pixels and their respective neighbourhoods in the MR images. In this case, the target pixels are the ones belonging, respectively, to the thrombus and the outer wall boundaries manually selected on the MR image dataset. The overall outcomes of the training stage comprise a statistical shape model and two appearance models defining the inner and outer faces of the aortic wall: a texture model of the thrombus boundary and an intensity model of the outer wall boundary. The variables of each model will be explained in the following sections.

The segmentation stage includes all the processes applied when a new patient MRI-MRA dataset arrives (Figure 2). It starts with a semiautomatic segmentation of the vessel's lumen in the MR image with radiological contrast. The lumen contour initializes the thrombus boundary search, while the thrombus boundary initializes the outer wall boundary search. Boundary searches are performed in a slice-by-slice 2D manner, while the lumen segmentation is performed in 3D. Following sections explain more in detail the proposed methods.

#### 2.2.1. Shape Model

The shape model for AAA was computed using experts' delineations performed using 75 CTA studies. Once the statistical shape model is constructed, CTA images are no longer required for the segmentation process, and only the statistical parameters of the model are used to constrain the evolution of the deformable model.

Shapes are described by a set of landmark points which ideally denote the same geometrical points in different objects. Cootes et al. [20] defined the concept of point distribution model (PDM) for the use of landmarks as basis for a statistical shape model. PDM is composed of four processes: landmark selection, landmark alignment, landmark correspondence, and dimensionality reduction using principal component analysis (PCA).

Following the scheme defined by [21], a fixed number of slices (19) were interpolated between renal arteries and the iliac arteries. A fixed number of landmarks (20) were placed in each slice, equiangularly distributed along the contours that were drawn manually by an expert, for a total of 380 landmarks for each 3D shape. The starting point of each contour was the most anterior point (in the coronal axis) with the same sagittal coordinate as the contour's centre of mass.

Axis variations were omitted and only cross sections' variability was modelled. To this end, every shape was straightened by translating each contour such that its centre of mass was in the origin of coordinates. Axis variability was not computed in the shape model because the segmentation process was always initialized using the lumen extracted from MRA images. In this way, variations were limited to dilations and contractions.

PCA [22] was applied to reduce the dimensionality of the problem of axis variability and to select only the most significant variation modes for the shape description. PCA starts computing the mean shape of the set, identified as $\overset{-}{x}$, and the covariance matrix described by

(1) $$\begin{array}{c} S=\frac{1}{n-1}\overset{n}{\underset{i=1}{\sum }}\left(\;x_{i}-\overset{-}{x}\;\right){\left(\;x_{i}-\overset{-}{x}\;\right)}^{T}. \end{array}$$

The modes of variation of the set are given by the eigenvalues *λ*_*i*_ and the eigenvectors Φ_*i*_ of the covariance matrix *S*. Following this formulation, there are 3*∗*380 = 1140 modes of variation in a set of 380 three-dimensional points. However, some of these modes of variation are considerably more significant. We selected only the modes of variation that represent between 90% and 98% of the total variability, and considered that the rest of the modes induced negligible variations. Any allowed shape can be expressed by a linear combination of the mean shape and the principal modes of shape variation, as described in (2), where *x* is the represented shape, $\overset{-}{x}$ is the mean shape, Φ_*i*_ are the eigenvectors of the covariance matrix, *c* is the number of principal modes, and *b* is a vector containing the shape parameters that define plausible variations:

(2) $$\begin{array}{c} x=\overset{-}{x}+\overset{c}{\underset{i=1}{\sum }}b_{i}\Phi_{i}. \end{array}$$

Commonly, *b*_*i*_ are restricted to a plausibility interval of $\left[-3\sqrt{\lambda_{i}},3\sqrt{\lambda_{i}}\right]$. However, aiming to create plausible shapes and not independent restrictions for every parameter *b*_*i*_, a global verisimilitude limit was defined to act simultaneously over the *c* principal components, such that the Mahalanobis distance (*D*_*m*_) from the mean is less than a suitable value (3), as described in [15]. Consider

(3) $$\begin{array}{c} D_{m}=\overset{c}{\underset{i=1}{\sum }}\frac{b_{i}^{2}}{\lambda_{i}}\leq M_{t}, \end{array}$$

where *λ*_*i*_ are the eigenvalues associated with the Φ_*i*_ eigenvectors. If each shape parameter *b*_*i*_ is normally distributed, then *D*_*m*_ will be *χ* ^2^ distributed. As a result, *M*_*t*_ can be selected using the *χ* ^2^ distribution depending on the number of significant variation modes *c* (as degrees of freedom) and the desired statistical significance (*t*), to include a suitably large proportion of possible realizations.

If the parameters *b*_*i*_ do not satisfy the inequality expressed in (3), they are limited using a proportionality constant *α* which reduces the values of all the components *b*_*i*_ in a similar way until (4) is satisfied. Consider

(4) $$\begin{array}{c} \left(\;\overset{c}{\underset{i=1}{\sum }}\frac{{\left(\alpha b_{i}\right)}^{2}}{\lambda_{i}}\;\right)=M_{t}. \end{array}$$

Thus, the combination of *α*, *b*_*i*_, and Φ_*i*_ describes the principal modes of variation of the AAA cross sections and the shape restrictions applied to the deformable model.

#### 2.2.2. Appearance Model

Fitting the shape model to a new image requires a notion of object boundary appearance. To derive the boundary appearance from the training set, grey value profiles are sampled around each landmark. Due to the limited number of MR images, robust appearance characterization using traditional approaches is not possible. Thus, we followed the scheme proposed by [23], which postulated that it is possible to specify the boundary appearance, in an explicit manner, defining a set of parameters.

**Figure 3:**
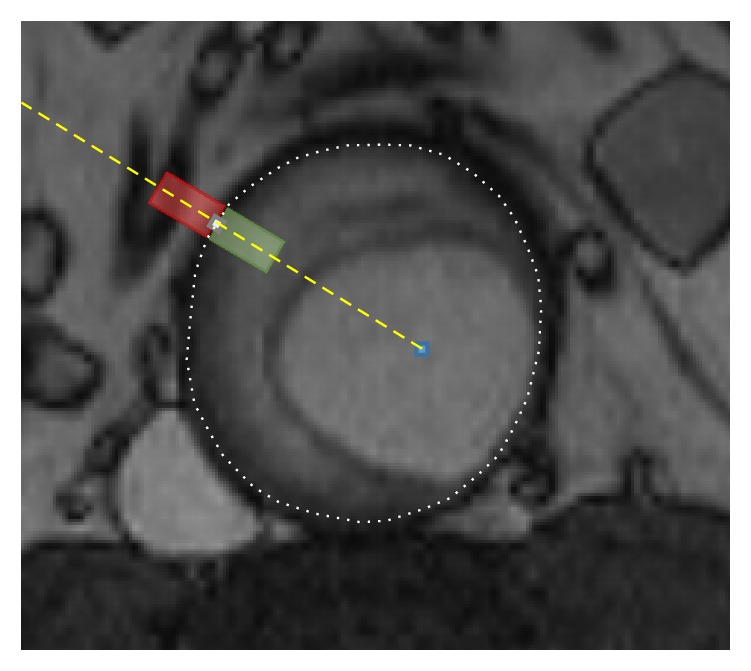
Example of the texture characterization. For every landmark on the manual delineation, a 11 × 3 mm region perpendicular to the contour and centred in the landmark is defined. The pixels of the region are labelled as “interior” or “exterior” depending on their relative position with the landmark location.

The main objective of this stage was to be able to discriminate between pixels belonging to the thrombus and pixels belonging to the aortic wall or adjacent external structures. Thus, a rectangular search region of 11 × 3 mm, centred in each landmark location and perpendicular to the manual delineation of the thrombus boundary, was used (Figure 3). Each pixel of the search region was categorized as interior (belonging to the thrombus, if the studied pixel is inside the manual delineation) or exterior (belonging to the vessel wall or surrounding structures, if the studied pixel is outside the manual delineation) and the differences between both groups were studied.

An exploration of the images allowed us to extract a set of patterns and, consequently, to define five variables trying to characterize the texture information of the thrombus boundary for every landmark surrounding:

- The region inside the vessel wall presents a higher mean intensity value than the regions comprising the vessel wall itself and the surrounding structures:
  1. Difference between mean intensities of the inner and outer regions: $\Delta I={\overset{-}{I}}_{\text{in}}-{\overset{-}{I}}_{\text{out}}$.
- The pixels belonging to the vessel wall, included in the exterior region, generally show significantly lower values than the adjacent pixels:
  1. Difference between minimum intensities of the inner and outer regions: Δ*m* = min(*I*_in_) − min(*I*_out_).
- Manual landmarks, located in the inner face of the vessel, present a higher intensity than the pixels belonging to the vessel wall, included in the external region:
  1. Difference between landmark intensity and minimum intensity of the outer region: *LmO* = *I*_landmark_ − min(*I*_out_).
- Pixels comprising the external region belong to the arterial wall or the adjacent structures. Those are structures with heterogeneous intensity values, so the standard deviation of the intensity values could be relevant:
  1. Standard deviation of the intensity in the outer region: std_out_ = *σ*_out_.
  2. Difference between the standard deviation of the intensities in the outer and inner regions: Δstd = *σ*_out_ − *σ*_in_.

These statistics should be normalized so that they can be combined in a single metric, defined such that the maximization of the sum of the normalized variables indicates the best possible position for the landmark. All the possible combinations using the proposed variables were tested in order to select the most suitable metric.

The outer wall boundary was estimated by searching a positive gradient from the thrombus boundary landmarks and in the outer direction. Moreover, a thickness limitation of 3 mm from the thrombus boundary was applied [24], restricting the misallocations in the regions where surrounding structures masked the vessel wall.

#### 2.2.3. Segmentation Stage

The segmentation process for a new patient only needs an MRI-MRA image study. The process starts with the aortic lumen segmentation on the MRA study of the patient using a 3D level set-based method [25]. The aortic lumen is differentiable due to the use of radiological contrast, so the user interaction is limited to the selection of a seed in the region of interest. The rest of the processes are completely automated.

Although MRI and MRA studies from the same patient had different image and voxel sizes, the physical coordinates were the same in both cases. Therefore, a simple rigid registration is performed using the DICOM information of each study. As a result, the centroid of the segmented lumen (using MRA) is included in the lumen structure in every slice of the MRI.

A circle centred in the cited centroid is used to initialize the search of the thrombus boundary using a 2D level set method. This initialization allows a texture-based refinement instead of a raw search, reducing the associated computing time. Once the initialization is performed, 20 landmarks are selected following the same strategy commented above in the shape model section.

A section of 11 × 3 mm, of which its longer sides were parallel to the line linking the landmark and the lumen centroid, was created around each landmark. The pixel of the region with the highest value for the sum of normalized intensity variables was selected. Once the intensity-based proposals for the landmarks' locations have been computed, the resulting shape is straightened according to the shape model proposed. After the shape restriction, in case the restriction is necessary, the straightening is undone. The intensity-based proposal and the shape-based restriction are iteratively performed until there are no modifications between iterations.

The thrombus boundary is used to initialize the outer wall boundary. This time the search is computed only outwards, limiting the search to a 3 × 3 mm area. The pixel with a larger positive gradient for every region associated with a landmark is selected. As in the case of the boundary selection, intensity-based estimation and shape restriction are both iteratively applied until no modifications between iterations appear.

### 2.3. Experiments

A set leave-one-out cross-validation experiment was designed and implemented due to the limitation of the image dataset's small size of 8 patients. One MRI-MRA patient study was excluded from the training dataset in each experiment and used as target image for the segmentation stage. So, while the training of the shape model was performed just once, the training of the appearance model was performed 8 times, using 7 MR studies each time. Thus, the independence between the training data and the test data is guaranteed.

The segmentation method is validated comparing the obtained delineation with manual delineations made by an expert, considered as ground truth. Two common metrics were used: Dice coefficient [26], defined following (5), and modified Hausdorff distance (MHD) [27] described by (6). Consider

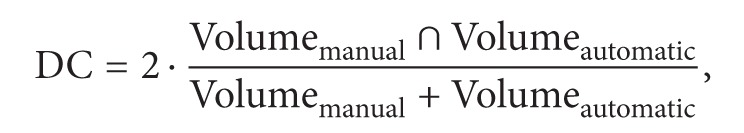
(5)

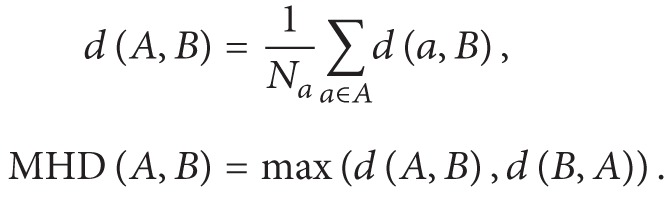
(6)MHD computes the distance between two 3D objects and improves the classical Hausdorff distance in two aspects: its value increments monotonically as the difference between the two objects increments, and it is robust against outliers.

## 3. Results and Discussion

### 3.1. Shape Model Tests

The ASM of the straightened transversal sections gather the variations of dilation along the abdominal aorta, mainly formed by the presence of the ILT. These are the relevant variation for our purpose since the segmentation is initialized by the lumen segmentation in each slice. Thus, the shape model is constructed to be independent of the evolutions of the centreline of the aorta and limiting the variability between different AAA shapes.

**Figure 4:**
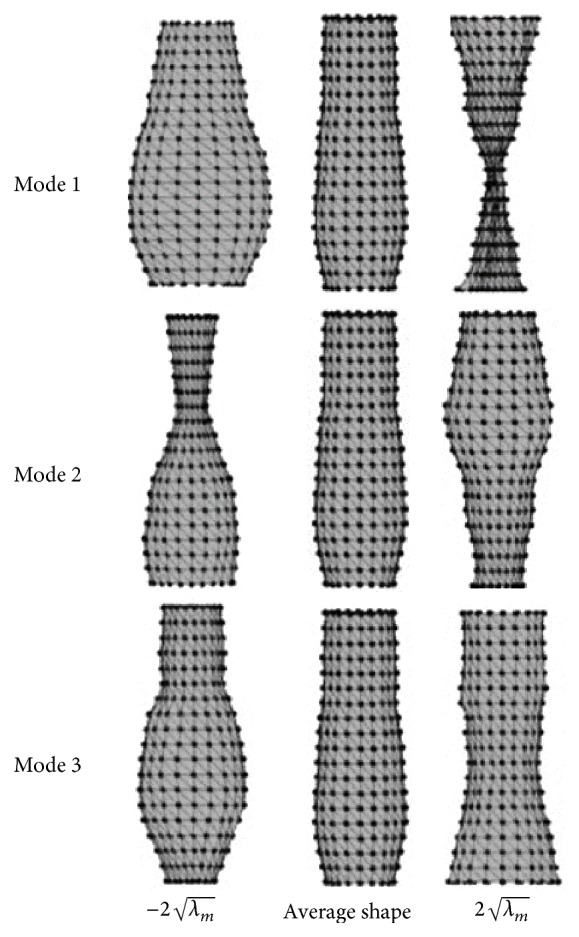
Significant variation modes of the transversal sections of the aorta, showing a variation of the average shape of $b=\pm 2\sqrt{\lambda_{m}}$.

After PCA, the results showed that 15 significant variation modes gathered 95% of the possible variations.  Figure 4 shows the three more significant variation modes, representing the variation defined by a value of $b=\pm 2\sqrt{\lambda_{m}}$ from the average shape.

Given *c* = 15 variation modes, different values in the range of *t* = [75%, 99%] were tested for the computation of the *M*_*t*_ threshold. Finally, we selected *t* = 90%, obtaining then *M*_*t*_ = 22.3. We observed that, allowing larger values of variability, the shape model resulted to be too flexible and obtained undesired delineations, especially in the regions where the initial location of the landmarks was displaced by the presence of adjacent structures. On the contrary, stronger restrictions of the variability resulted in a too rigid shape model, tending towards cylindrical shapes.

It is important to highlight that a large enough dataset could lead to very small changes in the shape model parameters when the patient dataset is extended. Meanwhile, different datasets will result in different values of *c*, *t*, *M*_*t*_, *b*_*i*_, and Φ_*i*_ variables, but the selection process will always remain constant and repeatable.

### 3.2. Appearance Model Tests

Results summarized in Table 1 show that the best combination of texture statistics is the one excluding the values of standard deviations, variables which reduce the quality of the result. It appears that the difference between mean intensities is the most influent parameter. The rest of combinations of three or two statistics, as well as the individual use of each texture statistics, present worse results than the ones presented in Table 1.

Thus, for every search region, the optimal landmark position is the one that maximizes the value of *m* = Δ*I* + Δ*m* + *LmO*.

### 3.3. Qualitative Evaluation

**Figure 5:**
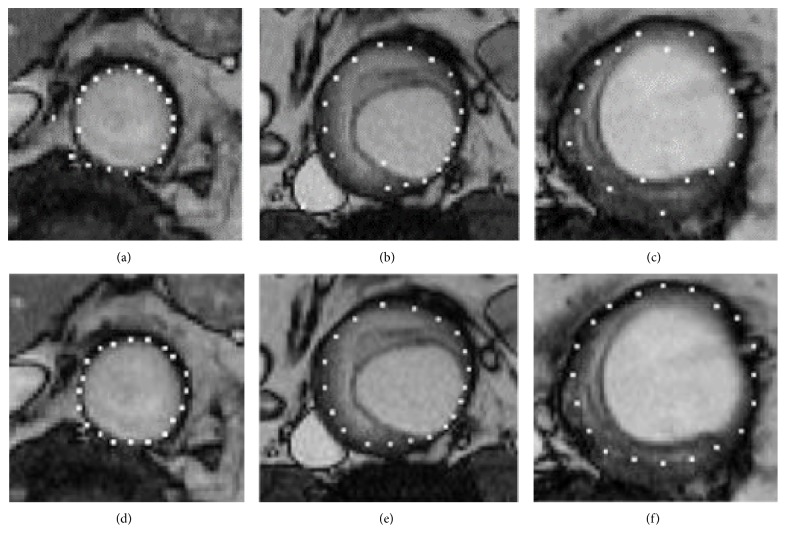
Thrombus boundary for different patients and slices: ((a)–(c)) wrong texture-based proposals; ((d)–(f)) shape-driven corrections.

In general, texture-based proposals are a good estimation in most cases. Nevertheless, some misestimations, due to changes of intensity in regions considered homogeneous or to adjacent structures that mask the target positions, can be found in some slices. The shape model modifies the landmark positions, adjusting them to an allowed point distribution. Isolated landmarks misestimated by the texture-based process (Figures 5(a)–5(c)) are corrected by the shape restrictions (Figures 5(d)–5(f)). Nevertheless, if the AAA shape is very specific and has not been included in the generation process of the shape model, the results can be overrestrictive.

**Figure 6:**
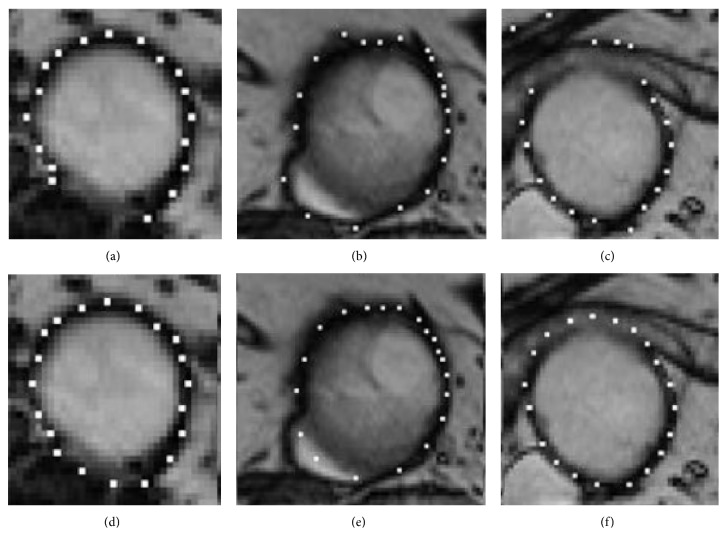
Outer wall boundary for different patients and slices: ((a)–(c)) wrong texture-based proposals; ((d)–(f)) shape-driven corrections.

Figure 6 illustrates different situations in the delineation of the outer wall boundary: adjacent structures with hypo- (left) or hyperintensities (middle) and blurred boundaries (right). In Figure 6(a) some landmarks are misallocated due to the presence of an adjacent structure with an intensity similar to the vessel wall one.  Figure 6(d) shows that the thickness limit and the shape restrictions improve qualitatively the results. Figures 6(b) and 6(c) display the incorrect landmarks positions due to adjacent structures with hyperintensity that, because of the partial volume effect, blurred the boundary. The thickness and shape restrictions limited the displacements and decrease the error but also restrict slightly some correct positions (Figures 6(e) and 6(f)).

### 3.4. Quantitative Evaluation

The performance of the segmentation method is quantitatively evaluated using manual delineations made by an expert, considered as ground truth.  Table 2 shows the values of volume overlap and MHD for the thrombus and outer wall boundaries, for the eight cases in the database. Mean values around 90% of volume overlap for both structures suggest a good agreement with an expert's manual delineations. Mean values for MHD slightly above 1 mm, compared with diameters around 25–30 mm of a healthy aorta [1], support this statement. It is important to emphasize that the shape model has been adjusted to be restrictive, so it displaces some correctly located landmarks, as shown in Figures 5 and 6.

Compared with the results reported by [13], we obtain very similar values of the modified Hausdorff distance for the outer wall boundary: 1.31 ± 0.62 mm versus 1.32 ± 0.32, reported in the commented article as Mean Distance. However, our results of the Dice coefficient are slightly lower, although close to 90% in most cases. Though Zohios et al. did not report a similar validation for the thrombus boundary since the process that they followed for the manual delineation discarded the more problematic regions. Moreover, manual delineation of the thrombus and vessel wall boundary using CT images is challenging due to the lack of contrast resolution, and the evaluation of the semiautomatic segmentation method is therefore affected by an uncertainty range.

**Figure 7:**
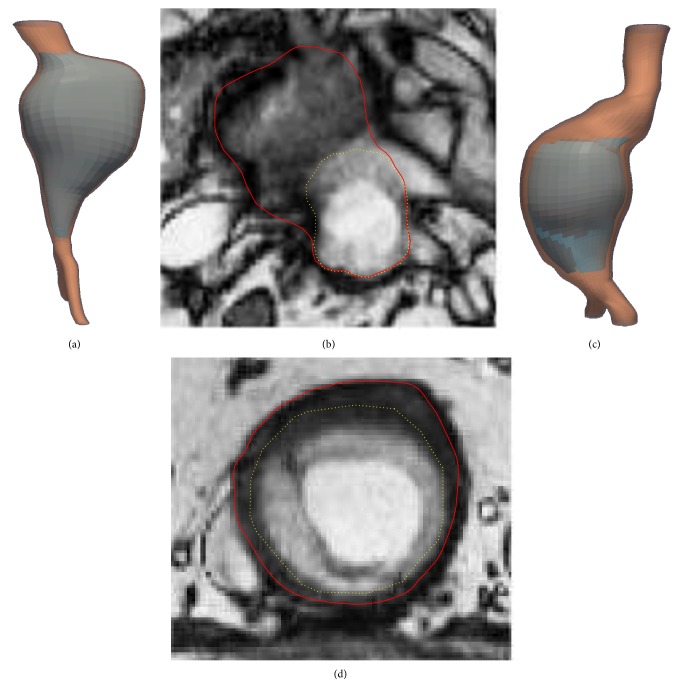
Patient 2 (top). Patient 6 (bottom). 3D reconstruction of aortic wall and ILT (left). MR slice with the manual (straight line) and automatic (dotted line) delineations (right).

It is important to highlight that mean values have been influenced and decreased by two specific cases with lower values of agreement: patient 2 and patient 6. Patient 2 has a specific geometry (Figure 7, top) not included in the shape model. Moreover, the abrupt dilation hinders the manual delineation, resulting in a large difference between the manual and the automatic delineations (Figure 7, top). Although shape model has been developed using 75 CT images, it has been demonstrated that it is necessary to use a larger number of cases in order to try to include as many different shapes as possible.

Patient 6 image shows a nonhomogenous ILT with 2 structures clearly differentiated (Figure 7, bottom). The automatic initialization performed using 2D level sets stops in the incorrect boundary and the texture proposal does not correct the contour. The incorrect initialization is performed in several slices, so the similarity values decrease notably. A better initialization step and/or a stop criterion for the thrombus boundary delineation, more robust against thrombus inhomogeneities, should lead to better results in this kind of cases.

The modular design of the proposed algorithm allows improvements modifying or adapting delimited sections of the method. Thus, the active shape model construction, the appearance model based on three statistical variables, the registration method using a rigid transform, or the initialization of the segmentation process based on a 2D level set method can be easily improved or adapted to other kinds of vascular imaging studies. Otherwise, the general scheme, using shape and texture information from contrasted and noncontrasted MR images to sequentially search lumen, thrombus, and outer wall boundaries, remains unmodified.

Manual delineation is a time-consuming task, so the evaluation has been performed using only one manual delineation as ground truth, following the same process as other previous works in the state of the art. Nevertheless, we know that manual delineations always entail a certain uncertainty [18], so future works are aimed at evaluating the proposed method's performance using several experts' delineations, taking into account the interspecialist variability.

## 4. Conclusions

A new method for segmenting human AAA thrombus and outer wall boundaries in MR multispectral studies has been presented. The modular design of the method combines shape and texture information, obtained from CTA and MRI image datasets, to guide a deformable model initialized by a level set-based segmentation.

The results show high correspondence between automatic and manual measurements for the vessel wall and thrombus areas. Resulting segmented images present a mean volume overlap with respect to manual segmentations of 88% and a mean modified Hausdorff distance of 1.14 mm for the thrombus boundary and 86% and 1.33 mm for the outer wall boundary. The use of the selected MR images allows better results for the thrombus boundary, maintaining good (although slightly lower than previously reported) values for the outer wall boundary.

While the dataset is small and further refinement is needed to make the method more robust against thrombus inhomogeneities, preliminary results of the segmentation of outer face of the vessel wall are similar to those in the literature, while improving substantially the results of the thrombus boundary.

## Figures and Tables

**Table 1:** Dice coefficient (%) between the manual and the automatic segmentations of the thrombus boundary of the 8 patient datasets for the different combination of texture statistics.

DC (%)	P1	P2	P3	P4	P5	P6	P7	P8	Mean
Δ*I* + Δ*m* + *LmO* + std_out_ + Δstd	89.7	81.5	91.0	89.8	86.2	82.0	88.5	88.8	87.2
Δ*I* + Δ*m* + *LmO* + std_out_	90.6	82.0	91.1	89.4	87.4	82.2	88.8	89.2	87.6
Δ*I* + Δ*m* + *LmO* + Δstd	90.3	82.3	91.3	89.3	86.2	82.0	88.2	89.2	87.3
Δ*I* + Δ*m* + std_out_ + Δstd	89.6	81.6	90.7	89.3	86.2	81.2	87.9	88.6	86.9
Δ*I* + *LmO* + std_out_ + Δstd	89.8	81.4	90.8	89.2	86.1	81.3	88.0	88.7	86.9
Δ*m* + *LmO* + std_out_ + Δstd	89.5	81.1	90.5	88.9	85.9	81.3	87.8	88.5	86.7
Δ*I* + Δ*m* + *LmO*	**90.8**	**82.4**	**91.3**	**89.9**	**87.6**	**82.8**	**89.3**	**89.9**	**88.0**

**Table 2:** Dice coefficient (DC) and modified Hausdorff distance (MHD), between the automatic delineations and the manual delineations performed by an expert, for the eight cases of the dataset and the two structures of interest.

	P1	P2	P3	P4	P5	P6	P7	P8	Av.	St. dev.
Thrombus boundary										
DC	0.91	0.82	0.91	0.90	0.88	0.83	0.89	0.90	0.88	0.03
MHD (mm)	0.88	1.91	0.76	1.24	0.94	1.29	1.28	0.86	1.14	0.37
Outer wall boundary										
DC	0.89	0.81	0.90	0.89	0.87	0.83	0.86	0.87	0.86	0.03
MHD (mm)	0.90	2.28	0.79	1.33	0.77	2.35	1.37	0.92	1.31	0.62
